# Louisville Medical Institute

**Published:** 1840-11

**Authors:** 


LOUISVILLE MEDICAL INSTITUTE.

The Clinical Lecture-Room, of which we made mention, in our last number, as promising to add materially to the means of instruction already in possession of the Faculty of the Medical Institute, is now fully completed, and will be used by the Professors of Clinical Medicine and Surgery during the approaching term. The apartment is spacious enough to accommodate four hundred students, all of whom by the favorable arrangement of the seats will have a full view of patients undergoing examinations and operations.

The number of students now in the city authorises the belief, that the Class this season will be as large as that of the last. Y.

